# FLAIR vascular hyperintensities and 4D MR angiograms for the estimation of collateral blood flow in anterior cerebral artery ischemia

**DOI:** 10.1371/journal.pone.0172570

**Published:** 2017-02-24

**Authors:** Matthias Gawlitza, Johannes Böhme, Maté Maros, Donald Lobsien, Dominik Michalski, Christoph Groden, Karl-Titus Hoffmann, Alex Förster

**Affiliations:** 1 Department of Neuroradiology, University Hospital Leipzig, Leipzig, Germany; 2 Department of Neuroradiology, University Medical Center Mannheim, University of Heidelberg, Mannheim, Germany; 3 Department of Neurology, University Hospital Leipzig, Leipzig, Germany; Heinrich-Heine-Universitat Dusseldorf, GERMANY

## Abstract

**Purpose:**

To assess FLAIR vascular hyperintensities (FVH) and dynamic (4D) angiograms derived from perfusion raw data as proposed magnetic resonance (MR) imaging markers of leptomeningeal collateral circulation in patients with ischemia in the territory of the anterior cerebral artery (ACA).

**Methods:**

Forty patients from two tertiary care university hospitals were included. Infarct volumes and perfusion deficits were manually measured on DWI images and TTP maps, respectively. FVH and collateral flow on 4D MR angiograms were assessed and graded as previously specified.

**Results:**

Forty-one hemispheres were affected. Mean DWI lesion volume was 8.2 (± 13.9; range 0–76.9) ml, mean TTP lesion volume was 24.5 (± 17.2, range 0–76.7) ml. FVH were observed in 26/41 (63.4%) hemispheres. Significant correlations were detected between FVH and TTP lesion volume (ρ = 0.4; *P*<0.01) absolute (ρ = 0.37; *P*<0.05) and relative mismatch volume (ρ = 0.35; *P*<0.05). The modified ASITN/SIR score correlated inversely with DWI lesion volume (ρ = -0.58; *P*<0.01) and positively with relative mismatch (ρ = 0.29; *P*< 0.05). ANOVA of the ASITN/SIR score revealed significant inter-group differences for DWI (*P*<0.001) and TTP lesion volumes (*P*<0.05). No correlation was observed between FVH scores and modified ASITH/SIR scores (ρ = -0.16; *P* = 0.32).

**Conclusions:**

FVH and flow patterns on 4D MR angiograms are markers of perfusion deficits and tissue at risk. As both methods did not show a correlation between each other, they seem to provide complimentary instead of redundant information. Previously shown evidence for the meaning of these specific MR signs in internal carotid and middle cerebral artery stroke seems to be transferrable to ischemic stroke in the ACA territory.

## Introduction

Acute ischemic stroke in the territory of the anterior cerebral artery (ACA) is a rare subtype of stroke which accounts for only approximately 2% of all ischemic strokes [1–3]. Since current strategies in stroke therapy mainly focus on recanalizing approaches targeting the internal carotid artery and the middle cerebral artery [4–8], the medical literature is sparse regarding neuroimaging procedures or even image-guided treatment options, respectively, for acute ischemic stroke particularly in the territory of the ACA [9]; the same holds true for the evaluation of collateral blood flow with novel imaging techniques in this stroke subtype, which might help decision making during the early phase of stroke. Anastomoses between the ACA and the posterior cerebral artery (PCA) and middle cerebral artery territory (MCA) as well as between the distal branches of the ACA have been described in detail in pathoanatomical studies [10]. However, the latter have been observed only infrequently, anastomoses between ACA and PCA may be absent [10] and even anastomoses between ACA and MCA may be insufficient to sustain a collateral blood flow to the ACA territory [11–13]. Thus, knowledge of individual differences in collateral blood flow might be crucial in treatment decisions in acute ACA infarction.

In 1999, vascular hyperintensities on fluid attenuated inversion recovery (FLAIR) images were first described in a series of patients with acute and subacute stroke [14] and termed "FLAIR vascular hyperintensity" (FVH). "Hyperintense vessel sign" is another term frequently used in the literature to describe this finding [15]. An association with acute large artery occlusion and chronic artery stenosis had been demonstrated [14,16,17] and a comparison of FLAIR images and conventional angiograms in patients with large vessel occlusion revealed that FVH was typically present in areas with retrograde collateral blood flow [18]. Meanwhile, most authors agree that proximal FVH is related either to slow anterograde blood flow or luminal thrombus, whereas distal FVH most likely represents retrograde collateral flow from arteries unaffected by occlusion [19,20].

Another innovative approach for the assessment of collateral blood flow has been introduced by Campbell and co-workers in 2013: 4D MR angiograms derived from DSC perfusion raw images [6]. A comparison of these 4D angiograms and conventional angiography demonstrated a very good correlation of collateral blood flow grades obtained separately with both methods [21]. By now, they have been used in only very few studies with middle cerebral artery (MCA) occlusion [6,21,22], in posterior circulation stroke [23,24] and in lacunar infarction [25]. To date, no study reported on FVH or 4D MR angiograms specifically in patients with ischemia in the ACA territory.

Overall, the existing literature provides growing evidence that FVH and 4D MR angiography might be useful techniques to capture stroke-induced perfusion deficits with consecutive tissue at risk, potentially allowing enhanced patient selection for recanalizing therapies. The present study is the first to systematically compare patterns of collateral blood flow in anterior cerebral artery ischemia with these two different approaches: (1) presence and extent of FVH and (2) extent of collateralization on 4D MR angiograms generated from perfusion-weighted raw images. Moreover, the study aims to assess whether these two markers of collateral circulation provide redundant or complimentary information regarding initial DWI lesion volume and the amount of hypoperfused and penumbral brain tissue.

## Material and methods

### Patients

After screening two hospital-based MRI databases, located at the University Hospital Leipzig and the University Hospital Mannheim (2005–2013), we identified 40 patients with acute focal cerebral ischemia in the territory of the ACA as indicated by an altered diffusion-weighted imaging (DWI) and/or perfusion-weighted imaging (PWI) in the respective territory, undergoing a standard stroke MRI protocol at hospital admission as well as during the hospital stay. All patients with the following criteria were eligible for the study: (1) technically adequate standard stroke MRI including PWI performed (2) within 12 hours after onset of symptoms, and (3) acute territorial infarction in the territory of the ACA. The demographic details, clinical presentation, and acute treatment were abstracted from the case records. The study was approved by the local ethics committees and did not require specific patient consent due to its retrospective nature.

### MRI studies

Magnetic resonance imaging was performed on 1.5-T MR systems (Magnetom Sonata, Symphony or Avanto, Siemens Medical Systems, Erlangen, Germany; Gyroscan Achieva or Intera, Philips Healthcare, Best, Netherlands) or on a 3T MR system (Siemens Trio, Siemens Medical Systems, Erlangen, Germany). Site-specific standardized protocols were used in all patients including (1) transverse, coronal and sagittal localizing sequences followed by transverse oblique images aligned with the inferior borders of the corpus callosum (applied on sequences 2 to 5); (2) DWI; (3) FLAIR images; (4) 3D time-of-flight MR angiography (MRA) and (5) PWI following the first pass of contrast bolus through the brain. Dynamic susceptibility contrast perfusion-weighted imaging was acquired using a gradient-echo echo planar imaging.

### Post-processing

#### Perfusion maps

The post-processing of the perfusion-weighted raw images was performed by a specific software, Signal Processing In NMR (SPIN, The MRI Institute for Biomedial Research, Detroit, USA). Deconvolution with singular value decomposition (SVD) was used [26]. The position of the arterial input function (AIF) was automatically determined by using the maximum concentration (Cmax), TTP and first moment MTT (fMTT). The concentration-time curve for arteries has short fMTT, short TTP and high Cmax. Twenty voxels, which best fitted these properties were selected. Then the concentration-time curves of these voxels were averaged, smoothed and truncated to avoid the second pass effect of the tracer.

#### 4D MR angiograms

Furthermore, perfusion-weighted raw images were used to create a dynamic angiographic representation of blood flow as described recently [6,21]. For this purpose, the baseline prebolus image was subtracted from each frame of the raw perfusion data as in digital subtraction angiography by use of SPIN.

### MRI analysis

FLAIR images and 4D MR angiograms were analyzed by two independent raters (M.G. and A.F., with six/eight years of experience in neuroimaging) blinded to the other MRI sequences and clinical information. Cases with discrepancies were re-reviewed by both readers and discussed until a consensus was reached.

#### Perfusion and diffusion lesions

Acute lesions were noted on DWI images. The topography and the corresponding dominant arterial territory were determined according to the maps by Tatu *et al*. [27,28]. Lesion size was measured on DWI by a manually delineated ROI, summation of these areas in cm^2^ on each section and multiplication with the slice thickness (plus interslice gap), to determine the volume in cm^3^ by use of OsiriX, a multidimensional image navigation and display software [29]. Similarly, PWI lesions were measured on TTP maps. Furthermore, the following values were calculated from the volumetric data: mismatch volume (MV = TTP lesion volume–DWI lesion volume) and relative mismatch volume [rMV = (TTP lesion volume–DWI lesion volume) /DWI lesion volume] at admission. For the calculation of rMV, the lesion volume was set at 0.1 ml if no DWI lesion was evident in order to avoid a division by zero.

#### FLAIR vascular hyperintensities

FVH were defined as focal, tubular, or serpentine hyperintensity relative to gray matter in the subarachnoid space or extending into the brain parenchyma. FVH was subdivided in either proximal and/or distal, whereas proximal FVH sign was defined as vascular hyperintensity in the A2 segment of the ACA. Hyperintense vessels in the more distal (A3 and A4) branches of the ACA were classified as distal FVH sign. For proximal FVH sign, a dichotomized classification was used i.e. either absent (0 points) or present (1 point). Distal FVH sign was graded as either absent (0 points), subtle (1 point) or as prominent (2 points), depending on the number of hyperintense sulcal vessels and their identifiability. Finally, a FVH score ranging from 0 up to 3 points was calculated for each patient [23].

#### 4D MR angiograms

The quality of the collateral circulation was assessed using a modification of the American Society of Interventional and Therapeutic Neuroradiology/Society of Interventional Radiology (ASITN/SIR) Collateral Flow Grading System as established recently [6,21,23]: grade 0 (no collateral vessels visible), grade 1 (slow collateral blood flow to the periphery of the ischemic site with persistence of some of the defect), grade 2 (rapid collateral blood flow to the periphery of ischemic site with persistence of some of the defect), grade 3 (collateral blood flow with slow but complete angiographic blood flow of the ischemic bed by the late venous phase), and grade 4 (complete and rapid collateral blood flow to the vascular bed in the entire ischemic territory by retrograde perfusion). Rapid collateral blood flow was defined as flow appearing within the arterial phase of the perfusion study.

### Statistical analysis

Data analysis was performed with Microsoft Excel (Office Excel 2003, Microsoft, Redmond, Washington) and SPSS 20.0 (SPSS, Inc., Chicago IL). Data are presented as mean values with standard deviations and ranges. Mean values were compared applying a one-way-ANOVA and a Bonferroni post-hoc correction was added. Correlation analysis was carried out by calculating Spearman’s ρ. In order to exclude the influence of different MRI scanner sites, scanner types as well as lateralization we introduced a multivariate modeling approach within a linear mixed effect model framework with three different models including FVH score, collateralization grade or both as well as MRI scanner location (Leipzig or Mannheim), scanner types (Achieva, Vision/Sonata/Symphony, Avanto, or Trio), and lateralization (left, right, or both hemispheres). *P* < 0.05 was considered statistically significant.

## Results

Between December 2006 and December 2013, 40 patients were included, 17 of them in center 1 ([blinded]), and 23 of them in center 2 ([blinded]). In all but one patient, one hemisphere was affected and evaluated in our analysis. One patient showed a stroke in the ACA territories of both sides and for the purpose of this study, both hemispheres were considered separately. The left hemisphere was affected in 21 patients (52.5%). Mean patient age was 72.2 (± 14.4; 47–88) years, 21 patients were female (52.5%). Mean time from symptom onset to imaging was 340.3 (± 201.9; 126–920) minutes, 10 patients (25%) were admitted with a wake-up stroke or an unknown symptom onset (of less than 24 hours in all cases). Mean DWI lesion volume was 8.2 (± 13.9; range 0–76.9) ml, mean TTP lesion volume was 24.5 (± 17.2, range 0–76.7) ml. In three patients no TTP volume could be calculated, as TTP slowing was also present in the directly adjacent parts of the MCA territory. Two patients were excluded for the evaluation of the modified American Society of Interventional and Therapeutic Neuroradiology/Society of Interventional Radiology (ASITN/SIR) Collateral Flow Grading System; one of them showed no perfusion lesion in the ACA territory, for the other no dynamic maps could be calculated as perfusion raw data were missing.

**Fig 1:**
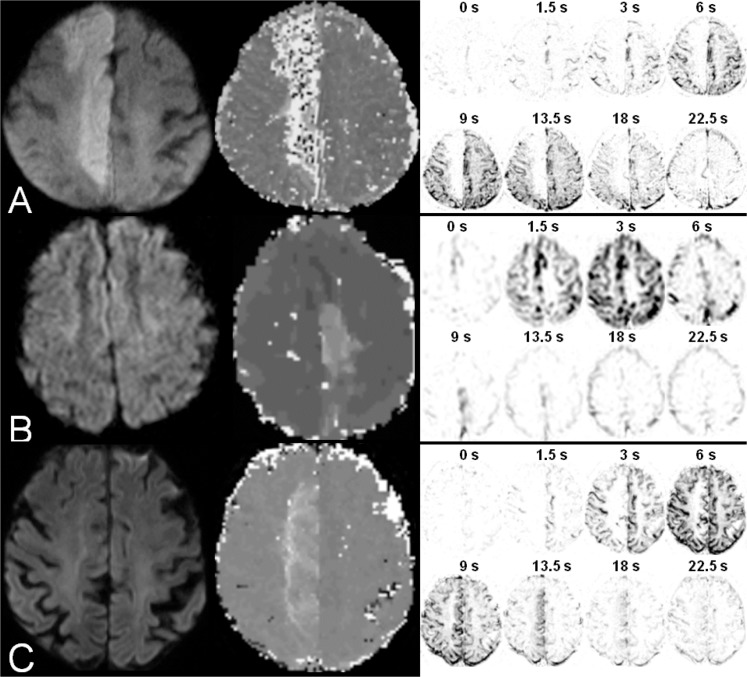
Examples of diffusion-weighted (left), corresponding perfusion-weighted images (middle), and collateralization grades on 4D MR angiograms (right) in anterior cerebral artery (ACA) occlusion. A. Grade 0 in a case of right anterior cerebral artery (ACA) occlusion. B. Grade 3 in a case of left ACA occlusion. C. Grade 4 in a case of right ACA occlusion.

**Fig 2:**
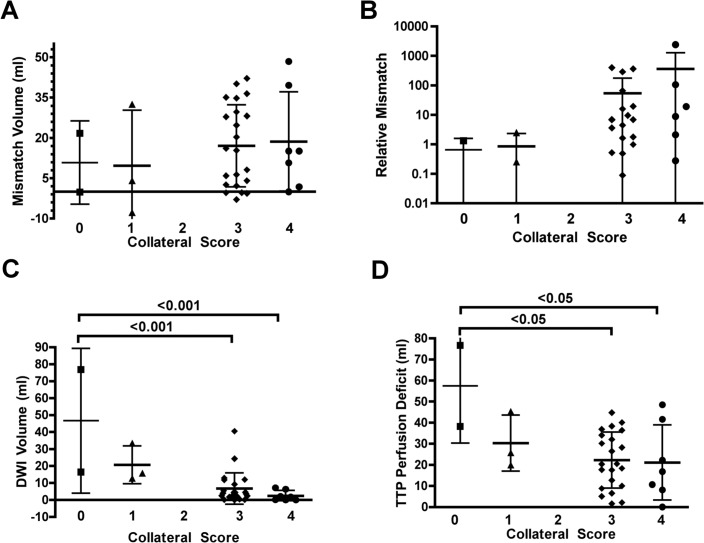
Relations between modified ASITN/SIR score and mismatch volume in ml (A); relative mismatch (B); DWI lesion volume in ml (C) and TTP lesion volume in ml (D). ANOVA with the modified ASITN/SIR score as a test variable revealed significant inter-group differences for DWI (*P*<0.001) and TTP lesion volume (*P*<0.05). The differences in DWI volumes were significant between scores of 0 and 3 as well as between 0 and 4 (both *P*<0.001; Fig 2C) applying the Bonferroni correction. TTP lesion volumes were significantly different between the same modified ASITN/SIR score combinations as above (both *P*<0.05; Fig 2D).

On 4D MR angiograms, 2/39 (5.1%) hemispheres showed no collateral blood flow from adjacent vascular territories. Collateralization was graded 1 in 5/39 (12.8%), 2 in 0/39 (0%), 3 in 24/39 (61.5%), and 4 in 8/39 (20.5%) hemispheres (for examples see Fig 1). For the modified ASITN/SIR score, a significant inverse correlation was observed with DWI lesion volume (ρ = -0.58; *P*<0.01) and also a tendency towards a significant negative correlation was observed with TTP lesion volume (ρ = -0.28; *P* = 0.05.) Moreover, the modified ASITN/SIR score showed a significant correlation with relative mismatch (ρ = 0.29; *P*< 0.05). In the multivariate modeling approach the correlations with DWI (p<0.01) and PWI lesion volume (p<0.05) remained significant. One-way ANOVA with the modified ASITN/SIR score as a test variable revealed significant inter-group differences for DWI (*P*<0.001) and TTP lesion volume (*P*<0.05). The Bonferroni correction proved that these differences in DWI lesion volumes were significant between scores of 0 and 3 as well as between 0 and 4 (both *P*<0.001; see Fig 2C). TTP lesion volumes were significantly different between the same modified ASITN/SIR score combinations as above, also applying the Bonferroni correction (both *P*<0.05; see Fig 2D).

**Fig 3:**
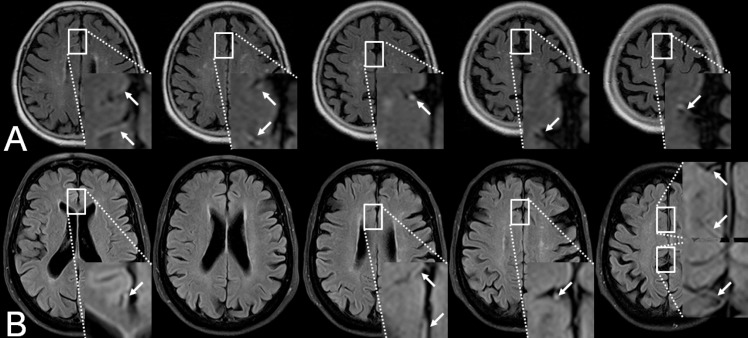
Two examples of FVH in the distal branches of the anterior cerebral artery (ACA, arrows) on FLAIR images in acute ischemic stroke due to ACA occlusion.

**Fig 4:**
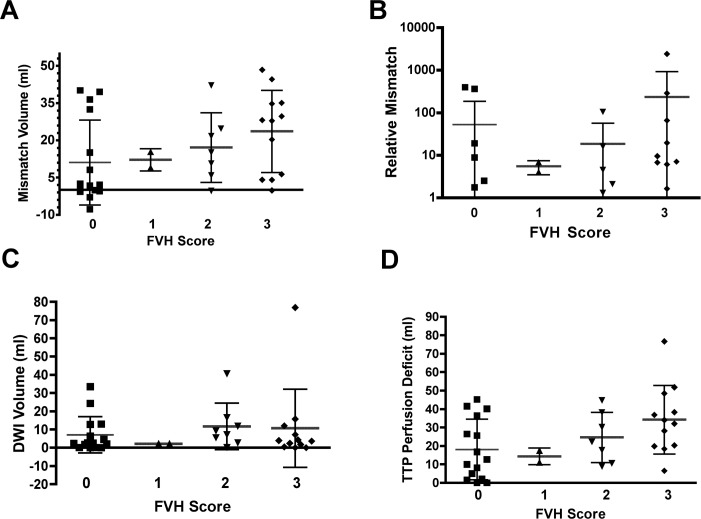
Relations between FVH score and mismatch volume in ml (A); relative mismatch (B); DWI lesion volume in ml (C) and TTP lesion volume in ml (D). ANOVA revealed no significant inter-group differences depending on FVH grade. However, a trend towards statistical significance was observed for TTP lesion volumes (*P* = 0.08).

FVH were observed in 26/41 (63.4%) affected hemispheres (for examples see Fig 3). Of these, 4/41 (9.8%) showed a score of 1, 10/41 (24.4%) a score of 2 and 12/41 (29.3%) a score of 3. Significant correlations were detected with TTP lesion volume (ρ = 0.4; *P*<0.01), absolute (ρ = 0.37; *P*<0.05) and relative mismatch volume (ρ = 0.35; *P*<0.05). In the multivariate modeling approach the correlation with PWI lesion volume (p<0.05) remained significant while no significant correlation with DWI lesion volume (p = 0.75) was found. Statistical significance was slightly missed for the ANOVA of FVH scores and differences in TTP lesion volumes (*P* = 0.08; Fig 4); this was accompanied by a tendency towards statistical significance comparing TTP lesion volumes of patients with FVH scores of 0 and 3 (*P* = 0.09).

No significant correlation was observed between FVH scores and modified ASITN/SIR scores (ρ = -0.16; *P* = 0.32).

## Discussion

In acute ischemic stroke due to large vessel occlusions, sufficient collateral blood flow is associated with improved clinical status on presentation and increased likelihood of recanalization with endovascular therapy [30]. Moreover, a favorable collateral circulation predicts a better functional patient outcome at 3 months [31]. In case of an ACA occlusion there are numerous possible collateral pathways from the ipsilateral MCA and PCA as well as from the contralateral ACA to provide oxygen to hypoperfused brain tissue [32]. Yet these pathways are known to show inter-individual differences. While conventional angiography is the gold standard for the assessment of leptomeningeal collateral flow, different non-invasive approaches for the assessment of collateral blood flow have been evaluated and used in the last years such as CT angiography [33,34], FLAIR images [15,23,35], T2* images [36], DSC-PWI [6,37,38], and arterial spin labeling perfusion-weighted imaging [39]. The rationale of the present work was to assess two proposed imaging markers of leptomeningeal collateral circulation in patients with an acute ischemic stroke in the ACA territory: FVH and 4D MR angiograms derived from DSC perfusion raw images.

To the best of our knowledge the present study is the first to describe that FVH occur frequently in the setting of an acute ischemic stroke in the ACA territory. While four studies investigated FVH in the posterior circulation [23,40–42], the majority of the published literature has been focusing on FVH within the scope of MCA ischemia [15,19,20,43,44]. In our patient series, more pronounced FVH were associated with larger TTP deficits, absolute and relative mismatch volumes but we observed no relation between FVH and baseline DWI lesion volumes. This corroborates previous evidence of FVH being an indicator of larger ischemic tissue fractions [15], whereas its value for the assessment of the infarct core itself seems to be limited. However, the fact that larger absolute and relative mismatch volumes were observed with more pronounced FVH underlines previous evidence that FVH are sensitive markers of PWI-DWI mismatch. In a recent work, Legrand and colleagues concluded that FVH in MCA occlusions contain “important hemodynamic information, assessable by the naked eye” and that they might be used to “identify patients with large PWI-DWI mismatches whenever perfusion data are missing” [44].

4D MR angiograms derived from DSC perfusion raw images were proposed as an imaging biomarker of collateral flow in 2013 [6]. This method had been investigated in a retrospective analysis of the EPITHET patient sample, mainly consisting of subjects with occlusions of the ICA and the MCA [6,45]. In accordance with the results described by Campbell and colleagues [6], a favorable collateral status correlated with relative mismatch in our patient sample. Moreover, we observed a negative correlation between collateral status and baseline DWI lesion volume meaning that favorable collaterals are associated with smaller infarct cores at the rime of initial imaging. Thus, our results contribute to the understanding of the fundamental importance of collateral flow for the existence of a significant amount of tissue-at risk in acute stroke. If a favorable collateral flow–which can be easily quantified by this specific technique–may be able to prevent or at least to decelerate early infarct growth in patients with acute ischemia in the ACA territory should be investigated in further studies.

The main limitations of the present study are its retrospective design and its small patient number, which can be mainly explained by the rare occurrence of the investigated stroke subtype. Our results are also limited by the fact that due to the configuration of the time of flight-angiography sequences (which cover the basal parts of the brain circulation with a focus on the circle of Willis) distal vessel occlusions were not confirmable in our patients; perfusion and diffusion lesions in the ACA territory were therefore used as a surrogate of the latter. Another limitation is that MR images were acquired on different scanner types (including 1.5T and 3T) in two different tertiary care university hospitals; it is yet unknown in which way different field strengths and scanner types influence the occurrence and the grade of FVH. Thus, definite conclusions should be drawn with caution and further studies on larger patient collectives seem justified.

In conclusion, the present study focused on two non-invasive methods for the estimation of cerebral collateral blood flow–FVH and 4D MR angiograms–and demonstrated that previous evidence, derived mainly from patients with ICA and MCA stroke seems to be transferrable to patients with an acute ischemic stroke in the ACA territory and not only to patients with ischemia in the posterior circulation. FVH and flow patterns on 4D MR angiograms are markers of perfusion deficits and tissue at risk. Whereas pronounced FVH can indicate larger perfusion lesions and mismatch volumes, favorable collaterals on 4D MR angiograms are strongly associated with small infarct cores. Moreover, as both methods did not show a correlation with each other, they seem to provide complimentary instead of redundant information. Future research is needed to verify if these specific information about collateral blood flow could be used to improve patient selection for recanalization therapies or to extend the therapeutic time window in acute ischemic stroke.
